# Cracks in the foundation: disrupted epistemic trust as a link between trauma, PTSD and disturbances in self-organization

**DOI:** 10.1038/s41398-026-04442-3

**Published:** 2026-09-17

**Authors:** Eileen Lashani, Peter Fonagy, David Riedl, Hanna Kampling, Johannes Kruse, Astrid Lampe, Veronika Engert, P. Read Montague, Elmar Brähler, Jörg M. Fegert, Cedric Sachser, Tobias Nolte

**Affiliations:** 1 Institute of Psychosocial Medicine, Jena University Hospital, Jena, Germany; 2 Anna Freud, London, United Kingdom; 3 Research Department of Clinical, Educational and Health Psychology, UCL, London, United Kingdom; 4 University Hospital of Psychiatry II, Department of Psychiatry, Psychotherapy and Psychosomatics, Medical University of Innsbruck, Innsbruck, Austria; 5 Ludwig Boltzmann Institute for Rehabilitation Research, Vienna, Austria; 6 Department of Psychosomatic Medicine and Psychotherapy, Justus Liebig University Giessen, Giessen, Germany; 7 Department for Psychosomatic Medicine and Psychotherapy, Medical Center of the Philipps University Marburg, Marburg, Germany; 8 VAMED Rehabilitation Center, Schruns, Austria; 9 German Center for Mental Health (DZPG), Halle-Jena-Magdeburg, Germany; 10 Center for Intervention and Research in adaptive and maladaptive brain Circuits underlying mental health (C-I-R-C), Halle-Jena-Magdeburg, Germany; 11 Centre for Human Neuroscience Research, Virginia Tech, Blacksburg, VA USA; 12 Department of Psychosomatic Medicine and Psychotherapy, University Medical Center of the Johannes Gutenberg University Mainz, Mainz, Germany; 13 Clinic and Polyclinic for Psychosomatic Medicine and Psychotherapy, Rostock University Medical Center, Rostock, Germany; 14 Department of Child and Adolescent Psychiatry/Psychotherapy, University of Ulm, Ulm, Germany; 15 German Center for Mental Health (DZPG), Mannheim-Heidelberg-Ulm, Germany; 16 Department of Clinical Child and Adolescent Psychology, Otto Friedrich University Bamberg, Bamberg, Germany

**Keywords:** Psychiatric disorders, Human behaviour, Pathogenesis

## Abstract

Traumatic experiences profoundly impact mental and physical health, alongside an individual’s trust in their social environment. Disruptions in epistemic trust – the capacity to evaluate and trust socially communicated information – are theorized to increase vulnerability to psychopathology by impairing social learning. This cross-sectional study investigated associations between trauma characteristics and epistemic stance using self-report data from a representative German sample (*N* = 2 519). We examined relationships among trauma type, age at trauma, epistemic stance, PTSD and cPTSD. Neither trauma type nor timing alone predicted epistemic stance. However, their interaction revealed that early relational trauma demonstrated the strongest association with epistemic disruption. Within epistemic stance, heightened credulity mediated associations between trauma and both PTSD and cPTSD symptoms. These findings align with the hypothesis that the association between trauma exposure and epistemic stance depends on trauma type and timing, with early relational trauma showing the strongest links to epistemic disruption and related psychopathology. This differentiated perspective on trauma characteristics highlights epistemic stance as a potential mechanism that may advance developmental models of psychopathology and future clinical research.

## Introduction

Traumatic events exert far-reaching effects on mental and physical health [1–3]. Childhood trauma, in particular, has profound implications for psychosocial development and is strongly associated with increased lifetime vulnerability to psychiatric disorders [4–6]. One of the most direct and debilitating outcomes of trauma exposure is post-traumatic stress disorder (PTSD), characterized by symptoms of re-experiencing, avoidance, negative affect, and hyperarousal [7]. Closely related, complex PTSD (cPTSD) arises predominantly following severe, prolonged, or repetitive traumatization [8]. CPTSD encompasses PTSD symptoms but additionally features disturbances in self-organization (DSO), reflecting impairments in affect regulation, negative self-concept, and relational difficulties [8]. Thus, whereas PTSD symptoms primarily capture fear-based responses, DSO reflects broader and more pervasive trauma-induced disruptions in personality functioning (i.e., core self- and interpersonal capacities).

Although over 70% of individuals worldwide experience traumatic events, only a minority develop trauma-related psychopathology [9, 10]. This discrepancy suggests that individual resilience, vulnerability, and trauma characteristics significantly modulate the risk for PTSD and cPTSD, including its defining DSO features. The current study investigates contextual and individual factors associated with PTSD and DSO development, with a particular focus on epistemic stance as one potential mediator linking self-reported trauma to symptom severity.

Epistemic stance is a construct that has garnered increasing attention over the past decade. It refers to how individuals appraise and respond to socially communicated information [11]. Campbell and colleagues [11] delineate three overlapping facets that constitute the epistemic stance: epistemic trust, epistemic mistrust, and epistemic credulity. Epistemic trust describes an individual’s capacity to appraise socially communicated information as authentic, generalizable, and personally relevant [11], enabling selective and adaptive engagement with social learning opportunities – for example, being open to feedback from friends or clinicians when such openness is warranted. Epistemic mistrust denotes generalized skepticism or rejection of socially conveyed information, viewing interpersonal communication as untrustworthy or malevolent; a person might, for instance, view benign advice as manipulative or otherwise ill-intended and dismiss it. Epistemic credulity involves difficulty evaluating the reliability of information along with a compromised self-position, leaving individuals vulnerable to misinformation or exploitation. Credulous individuals may, for example, readily accept contradictory or harmful suggestions from untrustworthy others without appropriate discrimination. While the behavioral expressions of epistemic mistrust and credulity may seem like opposite poles of a single dimension, they represent distinct yet co-occurring strategies to navigate the social environment when adaptive epistemic trust is impaired [11].

The epistemic stance emerges within primary attachment relationships during early childhood [12, 13] – a critical developmental window during which trauma can be especially disruptive. Beyond its impact on biophysiological maturation and calibration [14–16], this period is vital for establishing social cognition, including mentalizing capacities (the ability to understand one’s own and others’ behavior in terms of underlying intentional mental states), attachment security, and social learning, particularly epistemic trust [12, 13, 17–19]. Epistemic trust is closely linked theoretically [12, 13] and empirically [11, 20, 21] to attachment security and effective mentalizing. Secure attachment relationships provide the interpersonal context in which children learn to interpret others’ intentions as reliable and relevant, thereby supporting the development of mentalizing capacities [12]. Epistemic trust can be understood as an extension of these experiences from the dyad to the broader social environment, fostering openness to socially communicated knowledge [13, 22]. While disruptions in attachment and mentalizing consistently mediate the negative impact of childhood maltreatment [23–27], recent theoretical work highlights disrupted epistemic trust as a critical third mechanism within this developmental psychopathology triad [18, 19, 28–30]. Fonagy and Campbell [31] (p. 5) assert that “many, if not all, types of psychopathology might be characterized by temporary or permanent disruption of epistemic trust and the social learning process it enables”.

Assuming this perspective, epistemic trust represents a critical source of resilience protecting against the development and persistence of psychopathology by facilitating social learning and enabling individuals to benefit from interpersonal relationships [13, 19, 30]. Childhood relational trauma – referring to adverse experiences within relevant attachment relationships – can disrupt epistemic trust formation. This may occur in the context of intentional harm (e.g., caregiver maltreatment) as well as non-intentional relational rupture (e.g., caregiver loss), both of which can undermine early relational security. Children exposed to unreliable, neglectful, or malevolent caregiving may become uncertain or fearful regarding others’ intentions, adopting a hypervigilant stance of mistrust or an indiscriminate stance of credulity [11, 28]. Cross-sectional evidence supports this conceptualization, demonstrating negative associations between childhood adversity and epistemic trust, alongside robust positive associations with epistemic mistrust and credulity [11, 20, 32]. In contrast to relational trauma, non-relational traumatic events that do not involve trust violations by close others – such as accidents, illness or natural disasters – may more directly evoke fear-based responses, without affecting epistemic trust and associated social cognition. However, research contrasting the effects of relational and non-relational trauma on the epistemic stance remains scarce.

Although heightened mistrust and credulity initially protect children in threatening interpersonal environments, they may become maladaptive across development [13, 19, 30]. Dysfunctional epistemic stances impede the growth of effective mentalizing capacities and distort social perception. Trust violations through relational trauma can therefore induce lasting alterations in an individual’s acceptance of information from social agents, including friends, partners, teachers, and therapists [12, 28]. Consequently, initially developmentally adaptive epistemic stances have profound implications for psychopathology if they become generalized and remain unrevised: they undermine social learning and relational security, thereby compromising affect regulation, self-concept, and interpersonal functioning – core features of DSO [32, 33]. DSO, in turn, may constitute a perpetuating risk factor for further psychopathology by predisposing individuals to emotional dysregulation and ineffective coping across development [34, 35]. DSO may therefore not only represent a consequence of early relational trauma and epistemic stance disruptions, but also co-occur with and confer further vulnerability to fear-based PTSD symptoms. Accordingly, epistemic mistrust and credulity have been linked not only to elevated DSO and overall impaired personality functioning, but also to symptoms of PTSD, depression, anxiety, and somatization [11, 20, 32, 36, 37].

Using this developmental framework, there is preliminary evidence that epistemic disruption acts as a mediator between childhood adversity and psychopathology [11, 20, 21], specifically concerning PTSD and DSO symptoms [32, 37]. However, these studies are limited by their cross-sectional design and reliance on general assessments of childhood trauma, often without differentiating specific trauma types or contrasting childhood trauma effects with those resulting from later-life experiences. Such comparisons represent a crucial next step in validating the developmental epistemic trust framework. Understanding which trauma characteristics are most predictive of epistemic stance disruptions is essential for identifying risk factors and improving trauma-informed interventions.

Prior research addressing trauma characteristics demonstrates that the developmental timing of traumatic experiences plays a critical moderating role [38, 39]. In line with the developmental perspective outlined before, the literature consistently supports childhood trauma as pivotal for cPTSD and associated DSO symptoms [40, 41]. Nevertheless, cPTSD may also develop following adult trauma, particularly of interpersonal nature [41, 42]. For PTSD, evidence suggests that individuals exposed to trauma earlier in life, particularly in early childhood, are not only more likely to develop symptoms, but also to experience subsequent traumatization [38, 43–45]. However, evidence on the timing effect remains inconsistent, with some studies finding no significant age-related differences [46, 47], and others reporting stronger PTSD associations with later trauma onset [48–50]. The biopsychosocial mechanisms underlying these heterogeneous findings are poorly understood [38]. Critically, studies frequently examine childhood adversity broadly, without adequately differentiating age-dependent effects of specific trauma types [38, 39].

Considering that age at trauma and trauma type may not be independent, Guina and colleagues [46] found that the first traumatic experience is most commonly of interpersonal nature. Within interpersonal trauma, literature consistently indicates that intentional trauma, i.e., harm deliberately inflicted by another person (e.g., physical or sexual abuse), is particularly likely to lead to PTSD [44, 51, 52] and cPTSD [41, 42]. Beyond such severe but less common trauma types, large-scale studies have identified the unexpected or traumatic death of a loved one – a non-intentional relational trauma – as a prominent cause of post-traumatic stress symptoms in general populations [44, 53, 54]. These findings suggest that not only intentional trauma, but also non-intentional relational ruptures significantly increase vulnerability to PTSD and DSO, especially when occurring early in life.

Taken together, age at exposure and trauma type demonstrate differential associations with PTSD and DSO symptomatology. This study aims to shed light on one of the potential mechanisms underlying these differences, proposing epistemic stance as one potential link between trauma characteristics and symptom severity. Specifically, this study aims to: 1) replicate, in a large representative community sample, previous findings supporting the epistemic trust framework of developmental psychopathology, whereby early relational adversity associates with disrupted epistemic stance and heightened psychopathology; and 2) substantiate this evidence by contrasting early relational trauma with non-relational trauma across the lifespan. To achieve this, we drew from a cross-sectional representative German population sample. We hypothesized that (H1) childhood trauma exposure and (H2) relational trauma would each be associated with reduced epistemic trust and heightened mistrust and credulity, relative to adulthood or non-relational trauma. Further, (H3) epistemic stance was expected to partially mediate the link between trauma exposure and symptom severity, such that lower trust and higher mistrust and credulity would predict greater PTSD and DSO symptoms. The hypotheses and methods of this study were preregistered at https://osf.io/cxvej.

## Method

### Participants

The present sample was drawn from a representative population survey conducted by the demographic consulting firm USUMA (Berlin, Germany) between December 2020 and March 2021. Data collection complied with the Declaration of Helsinki and the ethical guidelines of the ICC/ESOMAR Code. Ethical approval was granted by the Ethics Committee of the Medical Faculty, University of Leipzig (No. 474/20-ek).

Participants were randomly selected using the ADM (Arbeitskreis Deutscher Markt- und Sozialforschungsinstitute e.V.) area sampling framework, a probability-based sampling system designed to obtain representative samples of the German general population [37]. Inclusion criteria comprised age ≥ 16 years, fluency in German, and provision of written informed consent. For minors, consent was also obtained from a parent or legal guardian. Data were collected through face-to-face interviews and self-administered questionnaires, resulting in a final sample of *N* = 2 519 individuals (age range: 16 – 96 years; *M* = 50.33, *SD* = 18.06). The sample was 52.5% female, 47.4% male, and 0.2% identified as another gender. 77.3% had not completed higher education, while 21.9% had. Monthly household income was < 1 250€ for 15.2%, 1 250 – 2 500€ for 38.7%, and > 2 500€ for 46.1%. This sample has been described in greater detail in previous publications addressing different research questions [32, 37].

### Questionnaires

#### PTSD symptoms

To assess significant trauma and related psychopathology, the International Trauma Questionnaire (ITQ [55]) was administered. The ITQ first asks respondents to identify and briefly describe their most distressing traumatic life experience in an open-text field. All subsequent questions are answered with reference to this selected event. Comprising 18 items rated on a 5-point Likert scale (1 = “not at all”, 5 = “extremely”), the questionnaire evaluates PTSD (re-experiencing, avoidance, sense of current threat) and DSO symptom clusters (affective dysregulation, negative self-concept, relational difficulties). We employed dimensional scoring for PTSD and DSO subscales (six items each) rather than dichotomous diagnostic categories, to retain variability and to capture a broader range of trauma-related symptom expression in this non-clinical community sample. Internal consistencies were strong (PTSD: α = 0.89, ω = 0.94; DSO: α = 0.87, ω = 0.92).

#### Trauma characteristics

Trauma characteristics were derived from participants’ descriptions of the events they subjectively experienced and identified as their most salient traumatic event within the ITQ. This operationalization captures subjective traumatic salience rather than requiring objective corroboration of the events, consistent with prior research emphasizing the relevance of perceived trauma in predicting psychological outcomes [56–58]. Responses were first processed via a semi-automated R [59] script (available at https://osf.io/kfrez/), followed by manual review and refinement through two trained research assistants. To estimate interrater reliability, they independently coded a random subset of the data (20%), achieving excellent agreement (Cohen’s κ: 0.93 – 0.99). The full coding scheme including definitions, decision criteria, and examples for each category is detailed in Supplementary Table 1.

##### Age

Using participant’s current age and indicated recency of the traumatic experience (ranging from “less than 6 months ago” to “more than 20 years ago”), the age range during traumatization was deduced. Age at trauma was coded by categorizing events occurring before age 18 as *childhood* and later events as *adulthood*. Additionally, ages were categorized into age subgroups at trauma according to Erikson’s developmental stages [60], as displayed in Table 1. Due to low subgroup counts in some categories and based on minimum sample size requirements from an a priori power analysis, *infancy and early childhood* (< 6 years; *n* = 8) and *middle childhood* (7 to 12 years, *n* = 10) were merged into a single *childhood* category.

**Table 1:** Characterization of traumatic events.

	*n*	%		*n*	%
Presence of trauma			Trauma nature		
yes	1 281	50.9	relational	685	27.2
no	465	18.5	non-relational	329	13.1
not specified	773	30.7	not specified	267	10.6
Age at trauma					
childhood (≤18 years)	106	4.2	Trauma type		
			loss	512	20.3
adulthood (>18 years)	987	39.2	separation	91	3.6
			emotional violence	23	0.9
not specified	188	7.5	physical violence	25	1.0
Age subgroup at trauma			sexualized violence	34	1.3
childhood (≤12 years)	33	1.3	war/ terror	29	1.2
			natural disaster	17	0.7
adolescence (13–18 years)	40	1.6	accident	115	4.6
			illness/ surgery	168	6.7
young adulthood (19–40 years)	294	11.7	other	192	7.6
			not specified	75	3.0
middle adulthood (41–65 years)	410	16.3			
older adulthood (>65 years)	152	6.0			
not specified	352	14.0			

Percentages are referenced to the full sample size of *N* = 2 519. When answers were missing, ambiguous or lacked sufficient detail for categorization, they were coded “not specified”.

##### Trauma type

Trauma type was determined through manual coding of participants’ open-text descriptions, informed by the potentially traumatic life events listed in DSM-5 [4] (e.g., physical or sexual violence, accidents, natural disasters, war and terror) and broadened to include relational and attachment-relevant events relevant to the DSO symptom cluster described in ICD-11 [8] (e.g., emotional violence, separation). When participants reported trauma that was witnessed or learned about involving close others, these events were coded according to the thematic nature of the event itself, consistent with DSM-5 Criterion A definitions [4]. Events not fitting these classifications (e.g., COVID-19-related experiences, economic hardship, imprisonment) were coded as *other*. Given substantial heterogeneity, the *other* category was excluded from further analyses. The final categorization and their frequencies are presented in Table 1.

##### Trauma nature

Trauma types were dichotomized into *relational* and *non-relational*. Trauma was considered relational, when it involved adverse events occurring within interpersonal relationships (loss, separation, emotional, physical, sexualized violence), whereas non-relational trauma reflects events not primarily involving interpersonal bonds (war and terror, natural disaster, accident, illness and surgery).

Loss and separation represent highly distressing relational experiences, particularly when occurring at a young age, and may therefore relate to unmentalized and traumatically processed ruptures of attachment [17] and epistemic trust [12, 13]. However, they are not traditionally considered traumatic events unless unexpected, accidental, or violent [7]. To examine whether loss and separation function differently from other relational adversities, we additionally applied an *intentional* vs. *non-intentional* classification, where intentional trauma denotes deliberate harm inflicted by another person (emotional, physical, sexualized violence), and non-intentional trauma includes all remaining categories.

#### Epistemic stance

Epistemic stance was assessed using the German 12-item version of the Epistemic Trust, Mistrust and Credulity Questionnaire (ETMCQ [11]), selected due to superior validity compared to the original 15-item scale (demonstrated in the present sample [21]). The ETMCQ comprises three independent subscales: *trust* (five items*), mistrust* (three items), and *credulity* (four items). Items were rated on a 7-point Likert scale (1 = “strongly disagree” to 7 = “strongly agree”). Internal consistencies were good for trust (α = 0.81, ω = 0.86) and credulity (α = 0.81, ω = 0.87). Given the questionable internal consistency for mistrust (α = 0.65, ω = 0.66), interpretation of results involving this subscale should be made with caution.

### Analysis

Analyses were conducted in R [59] (version 4.4.0). The corresponding code along with raw data supporting the conclusions are publicly available at https://osf.io/kfrez/.

As preregistered, the main analyses included only variables of primary interest; subsequently, analyses were repeated controlling for age, gender, education, and income. This stepwise approach allowed us to assess robustness to potential confounding while preserving clarity and interpretability in the main text; covariate-adjusted models are reported in the Supplementary Materials due to their increased complexity and limited impact on substantive conclusions. Participants with missing values on relevant variables were excluded from the respective analyses. For all tests, two-tailed *p*-values < 0.05 were considered statistically significant.

In addition to preregistered analyses, exploratory analyses were conducted, including interaction effects of age at trauma and trauma nature on epistemic stance, a test of the full conceptual model, and sequential moderated mediation analyses. An alternative coding scheme for trauma nature was tested in supplementary analyses related to H2.

#### Analysis of variance

MANOVAs and ANOVAs were used to test construct validity and hypotheses H1 and H2. The multivariate approach allowed us to account for the expected interrelations of the three epistemic stance dimensions (trust, mistrust, and credulity), while preserving their conceptual distinctiveness. A construct check initially validated whether trauma presence was associated with epistemic stance (trust, mistrust, credulity), using a MANOVA. Criterion validity of the trauma coding was assessed with univariate ANOVAs predicting PTSD and DSO scores; convergent validity with the Adverse Childhood Experiences Questionnaire (ACE [61]) was similarly tested.

Hypothesis H1 was tested via two between-subjects MANOVAs with epistemic stance subscales as outcomes, comparing age groups (*childhood* vs. *adulthood* vs. *no trauma*) and, subsequently, detailed age subgroups. Likewise, H2 was tested with two separate MANOVAs using trauma nature (*relational* vs. *non-relational* vs. *no trauma*) and trauma type (detailed subgroups) as independent variables. An exploratory MANOVA combining H1 and H2 evaluated interactions between age group and trauma nature.

An a priori power analysis was conducted using G*Power [62] (version 3.1.9.6) to determine the required sample size for each MANOVA with three dependent variables and categorical independent variables with up to 10 levels. The analysis assumed a small-to-medium effect size (Cohen’s *f²[V]* = 0.0625), α = 0.05, and power (1 – β) = 0.90. The results indicated that a minimum of *n* = 17 per group would be necessary to detect a significant multivariate effect. Based on this, trauma age subgroups with insufficient sample sizes were combined to meet power requirements.

Prior to analysis, MANOVA assumptions were assessed (multivariate normality, homogeneity of covariance matrices, linearity, absence of multicollinearity, and independence of observations). Univariate (values deviating from group means by ≥ 3 *SD*s) and multivariate outliers (Mahalanobis distance at α = 0.001) for epistemic stance were identified and excluded (*n* = 30). Post-exclusion, visual inspection indicated no problematic assumption violations.

For all MANOVAs, univariate follow-up ANOVAs were evaluated only when the multivariate omnibus test was significant. Pairwise group comparisons provided Cohen’s *d* as effect sizes and were conducted using Tukey’s HSD, which controls the familywise error rate across all pairwise contrasts. No additional multiplicity correction was applied, consistent with recommendations for studies that do not test a single omnibus null hypothesis across models [63].

#### Mediation

Two mediation models tested the indirect associations of trauma presence (binary, derived from the ITQ) with PTSD and DSO symptoms via epistemic stance (trust, mistrust, credulity) (H3). Predictors were *z*-standardized to enhance interpretability and address potential multicollinearity. Indirect effects were tested with 5 000 bootstrapped resamples using the psych package for R [64] (version 2.4.6). They were deemed significant if their 95% CIs did not include zero. Given the cross-sectional data, these analyses are interpreted as tests of statistical associations consistent with the proposed theoretical model rather than as evidence of causal mediation.

#### Exploratory moderated mediation models

Conceptually, the full model assumes that early relational trauma exerts particularly detrimental effects on epistemic stance. To capture the interplay of trauma nature (r*elational* vs. *non-relational*) and developmental timing (*childhood* vs. *adulthood*) – rather than examining these characteristics independently – we conducted two exploratory moderated mediation analyses (PROCESS macro for R [65], Model 7), using either PTSD or DSO symptoms as outcomes. Continuous predictors were again *z*-standardized. Given that trauma characteristics only applied to participants endorsing trauma on the ITQ, analyses were limited to these individuals. Trauma nature served as the independent variable, with age group moderating the paths linking trauma nature to epistemic stance (trust, mistrust, credulity).

Additionally, in line with developmental accounts suggesting that disruptions in personality functioning may represent an adaptation to early relational adversity, we tested an exploratory sequential moderated mediation model. In this model, self-organization functions both as an outcome and as a mediator linking early adversity to trauma-related symptomatology. Specifically, we examined whether trauma characteristics (trauma nature and age group) predict DSO through epistemic stance, and whether DSO subsequently predicts PTSD symptoms. The model was implemented using custom code in PROCESS [65].

## Results

Descriptive statistics and correlations among study variables are summarized in Table 2. Results of the construct validity checks are reported in Supplementary Tables 2 and 3, with effects sizes detailed in Supplementary Note 1. In short, reporting traumatic events on the ITQ was associated with disrupted epistemic stance, particularly increased credulity. Additionally, disclosure of traumatic events appeared biased by epistemic stance, as participants withholding information showed reduced epistemic trust compared to those who disclosed. Overall, the trauma coding demonstrated good criterion validity with ITQ scores and convergent validity with ACE scores.

**Table 2:** Descriptive statistics and correlations of all included variables.

	*M*	*SD*	1	2	3	4	5	6	7	8	9	10
1. Trauma age												
2. PTSD	8.82	4.19	−0.04									
3. DSO	8.91	3.88	−0.09**	0.52**								
4. Trust	24.90	5.33	−0.02	−0.03	−0.18**							
5. Mistrust	10.97	3.67	0.02	0.16**	0.31**	−0.16**						
6. Credulity	11.99	5.10	0.04	0.24**	0.38**	−0.14**	0.57**					
7. ACE	0.90	1.71	−0.09**	0.30**	0.43**	−0.11**	0.18**	0.26**				
8. Age	50.33	18.06	0.45**	−0.00	0.01	−0.02	0.06**	0.00	0.00			
9. Sex (male)			0.02	−0.10**	−0.01	−0.14**	−0.02	−0.10**	−0.04	−0.01		
10. Education			0.03	−0.01	−0.05*	0.02	−0.05*	−0.16**	−0.07**	−0.11**	−0.00	
11. Income			−0.01	−0.10**	−0.18**	0.07**	−0.13**	−0.13**	−0.17**	−0.18**	−0.08**	0.16**

The table presents Pearson correlations among all study variables. *M* and *SD* represent mean and standard deviation. Trauma age refers to the age group at which trauma occurred, i.e., childhood or adulthood. The ITQ [54] was used to assess symptoms of PTSD and disturbances in self-organization (DSO). Epistemic trust, mistrust, and credulity were assessed with the ETMCQ [11]. Adverse childhood experiences were measured with the ACE [59]. * *p* < 0.05; ** *p* < 0.01; *** *p* < 0.001.

### H1: age at trauma

Results assessing effects of age at trauma are summarized in Table 3a. The MANOVA comparing traumatization in childhood, adulthood, or no trauma revealed significant differences, driven primarily by credulity: *F*(6,2668) = 12.90, *p* < 0.001, η_p_^2^ = 0.03. Specifically, participants reporting trauma during childhood (*d* = 0.31; 95% CI [0.07, 0.55]; *p* = 0.033) and adulthood (*d* = 0.43; 95% CI [0.31, 0.55]; *p* < 0.001) both exhibited higher credulity compared to non-traumatized participants. Contrary to our hypotheses, no significant differences emerged between childhood and adulthood trauma groups in epistemic trust (*d* = -0.11; 95% CI [-0.34, 0.12]; *p* = 0.602), mistrust (*d* = 0.06; 95% CI [-0.17, 0.29]; *p* = 0.868), or credulity (*d* = 0.12; 95% CI [-0.11, 0.35]; *p* = 0.553).

**Table 3:** Group differences in epistemic stance between age groups at trauma and trauma type.

a)	Trust	Mistrust	Credulity	b)	Trust	Mistrust	Credulity
Age at trauma				Trauma nature			
childhood (≤ 18 years)	26.1 (5.1)	10.9 (4.2)	12.1 (5.4)*	relational	25.4 (5.0)	10.9 (3.8)	12.6 (5.2)***
				non-relational	25.8 (5.0)	11.2 (3.9)	12.5 (5.2)***
adulthood (> 18 years)	25.5 (5.0)	11.1 (3.8)	12.7 (5.2)***	*no trauma*	*25.4 (4.9)*	*11.0 (3.5)*	*10.5 (5.0)*
					*F*(2,1428) = 0.84	*F*(2,1428) = 0.62	*F*(2,1428) = 24.61
*no trauma*	*25.4 (4.9)*	*11.0 (3.5)*	*10.5 (5.0)*				
	*F*(2,1335) = 0.67	*F*(2,1335) = 0.16	*F*(2,1335) = 26.49		*p* = 0.433	*p* = 0.539	*p* < 0.001
					η^2^ = 0.00	η^2^ = 0.00	η^2^ = 0.03
	*p* = 0.512	*p* = 0.852	*p* < 0.001				
	η^2^ = 0.00	η^2^ = 0.00	η^2^ = 0.04				
Age subgroup at trauma				Trauma type			
childhood (< 12 years)	25.6 (5.5)	11.7 (3.4)	11.3 (4.6)	loss	25.3 (5.0)	10.8 (3.7)	12.2 (5.1)***
				separation	25.7 (4.9)	10.8 (3.9)	13.0 (5.5)**
adolescence (13–18 years)	26.3 (5.6)	10.7 (4.1)	12.9 (5.7)	emotional violence	24.9 (5.3)	11.7 (3.9)	13.3 (4.7)
				physical violence	26.2 (5.5)	11.0 (3.8)	13.6 (4.3)
young adulthood (19–40 years)	25.8 (5.2)	11.1 (4.1)	13.2 (5.4)***	sexualized violence	25.9 (5.0)	12.6 (4.1)	15.4 (5.9)***
				war/ terror	25.4 (6.0)	11.0 (4.3)	11.6 (4.4)
middle adulthood (41–65 years)	25.3 (4.9)	11.3 (3.5)	12.8 (5.1)***	natural disaster	29.2 (4.9)	10.3 (3.4)	12.3 (4.4)
				accident	25.3 (4.9)	11.1 (4.1)	12.4 (5.6)*
older adulthood (> 65 years)	25.4 (4.7)	10.6 (3.8)	12.0 (5.2)*	illness/ surgery	25.8 (4.7)	11.4 (3.7)	12.7 (5.0)***
				*no trauma*	*25.4 (4.9)*	*11.0 (3.5)*	*10.5 (5.0)*
*no trauma*	*25.4 (4.9)*	*11.0 (3.5)*	*10.5 (5.0)*		*F*(9,1421) = 1.42	*F*(9,1421) = 1.28	*F*(9,1421) = 7.26
	*F*(5,1187) = 0.57	*F*(5,1187) = 0.89	*F*(5,1187)= 12.48				
					*p* = 0.172	*p* = 0.242	*p* < 0.001
	*p* = 0.721	*p* = 0.490	*p* < 0.001		η^2^ = 0.00	η^2^ = 0.00	η^2^ = 0.04
	η^2^ = 0.00	η^2^ = 0.00	η^2^ = 0.03				

Models were conducted with *n* = 1 338 (age groups), *n* = 1 193 (age subgroups), and *n* = 1 431 (trauma nature and type) participants. Descriptive values follow the structure *M* (*SD*). ANOVA results for each ETMCQ [11] subscale are reported below the descriptive values. Significant pairwise comparisons between each category and “no trauma”, based on Tukey-Kramer HSD tests, are indicated with asterisks: * *p* < 0.05; ** *p* < 0.01; *** *p* < 0.001.

When analyzing more refined age subgroups, we again observed a significant MANOVA: *F*(15,3561) = 6.06, *p* < 0.001, η_p_^2^ = 0.02. Contrary to our predictions, there were no significant differences among the age subgroups in epistemic stance beyond credulity. Specifically, credulity was significantly higher among participants reporting traumatic events during young adulthood (*d* = 0.54; 95% CI [0.38, 0.70]; *p* < 0.001), middle adulthood (*d* = 0.47; 95% CI [0.32, 0.61]; *p* < 0.001), and older adulthood (*d* = 0.31; 95% CI [0.11, 0.51]; *p* = 0.032), compared to those with no reported trauma. Participants who reported trauma during childhood or adolescence showed no significant differences in epistemic stance compared to those without trauma.

Including covariates (age, gender, education, income) did not alter the findings regarding age groups or subgroups at trauma (Supplementary Table 4). Given the surprising association between adulthood trauma and increased credulity, we exploratively examined whether trauma recency accounted for this finding. A multivariate linear regression indicated no significant effect of trauma recency on credulity (*b* = -0.17, *SE* = 0.11, *t*(1046) = -1.55, *p* = 0.122).

In summary, our hypothesis that earlier traumatization would be associated with greater disruption of epistemic stance was not supported. Nonetheless, credulity consistently emerged as the epistemic stance dimension most sensitive to self-reported trauma, irrespective of age at exposure.

### H2: type of trauma

Effects of trauma nature and trauma types on epistemic stance are summarized in Table 3b. The MANOVA examining trauma nature (*relational* vs. *non-relational*) revealed significant differences in epistemic stance, attributable primarily to credulity: *F*(6,2854) = 13.00, *p* < 0.001, η_p_^2^ = 0.03. Both relational trauma (*d* = 0.41; 95% CI [0.29, 0.53]; *p* < 0.001) and non-relational trauma (*d* = 0.40; 95% CI [0.25, 0.54]; *p* < 0.001) were associated with higher credulity compared to no trauma. However, relational and non-relational trauma did not differ significantly regarding epistemic stance.

When inspecting effects of the specific trauma types, significant differences in epistemic stance emerged, again driven by credulity: *F*(27,4263) = 4.07, *p* < 0.001, η_p_^2^ = 0.03. Specifically, credulity was highest among participants reporting sexualized violence, significantly exceeding scores for loss (*d* = 0.62; 95% CI [0.26, 0.97]; *p* = 0.018) and no trauma (*d* = 0.98; 95% CI [0.62, 1.34]; *p* < 0.001). Other trauma types were also associated with elevated credulity compared to no trauma, including loss (*d* = 0.34; 95% CI [0.21, 0.47]; *p* < 0.001), separation (*d* = 0.50; 95% CI [0.26, 0.73]; *p* = 0.001), accidents (*d* = 0.37; 95% CI [0.16, 0.58]; *p* = 0.017), illness and surgery (*d* = 0.45; 95% CI [0.27, 0.64]; *p* < 0.001).

Including covariates did not alter these patterns (Supplementary Table 4). Results using the exploratory *intentional* versus *non-intentional* trauma nature coding scheme are detailed in Supplementary Note 2 and Supplementary Table 5. Briefly, intentional trauma was significantly associated with increased credulity compared to both non-intentional trauma and no trauma.

In summary, different trauma types were differentially related to credulity but did not show associations with trust or mistrust. The hypothesis that relational trauma would be more detrimental to epistemic stance than non-relational trauma was not supported.

### Exploratory analysis: interactive effects between trauma characteristics

**Fig. 1:**
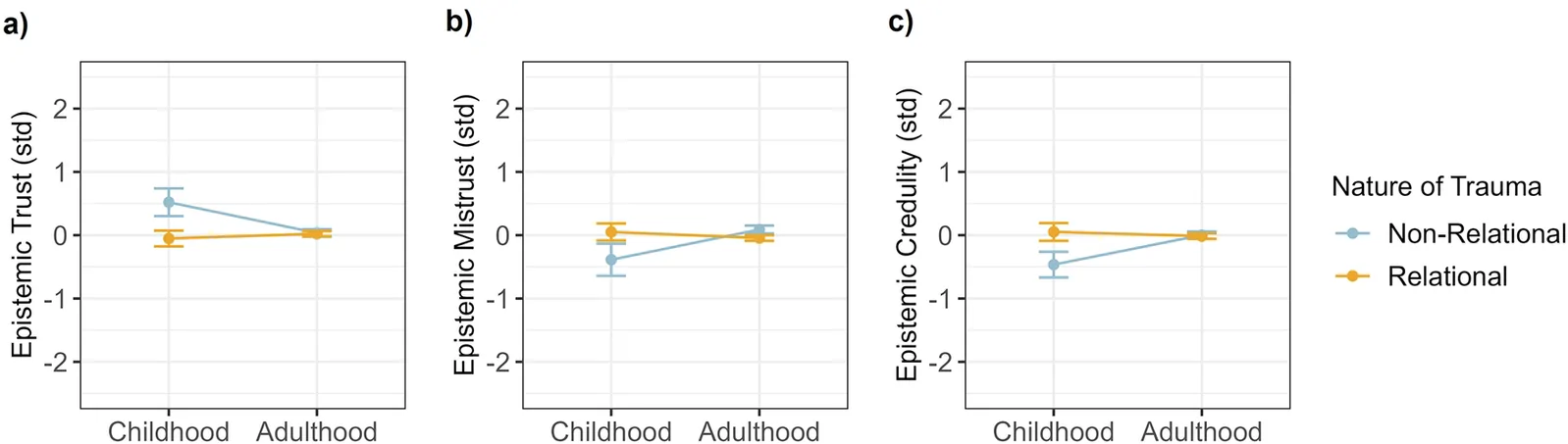
Interactions between nature of trauma and age at trauma. *Note*. Epistemic **a** trust, **b** mistrust, and **c** credulity were assessed with the ETMCQ [11]; each panel shows the corresponding outcome. Original subscales range from 5 to 35, 3 to 21, and 4 to 28, respectively. Scores were z-standardized for better comparability. Center values reflect the group means. Error bars represent the standard errors of the means. Childhood refers to ages ≤ 18 years, adulthood to ages > 18 years. The graphs are based on *n* = 852 participants.

To further elucidate how trauma characteristics jointly influence epistemic stance, we conducted an exploratory MANOVA testing the interaction between age at trauma and trauma nature. This interaction predicted epistemic stance (*F*[3,846] = 3.30, *p* = 0.020), with significant effects observed across all subscales: trust (*F*[1,848] = 5.75, *p* = 0.017), mistrust (*F*[1,848] = 4.76, *p* = 0.029), and credulity (*F*[1848] = 4.89, *p* = 0.027). Main effects of age and trauma nature remained non-significant. Figure 1 illustrates these interactions, consistently revealing less epistemic stance disruptions with non-relational compared to relational trauma in childhood; this pattern did not emerge when trauma occurred later in life.

Using the exploratory coding scheme (*intentional* vs. *non-intentional trauma*), no interaction effects emerged (Supplementary Note 2). This finding suggests that intentional trauma is associated with disrupted epistemic stance across all age groups, whereas broader relational trauma effects appear age-specific.

In summary, these exploratory findings highlight how trauma type and developmental timing interact in predicting epistemic stance disruptions, aligning with theoretical expectations.

### H3: mediation of trauma effects on PTSD symptoms across epistemic stance

**Fig. 2:**
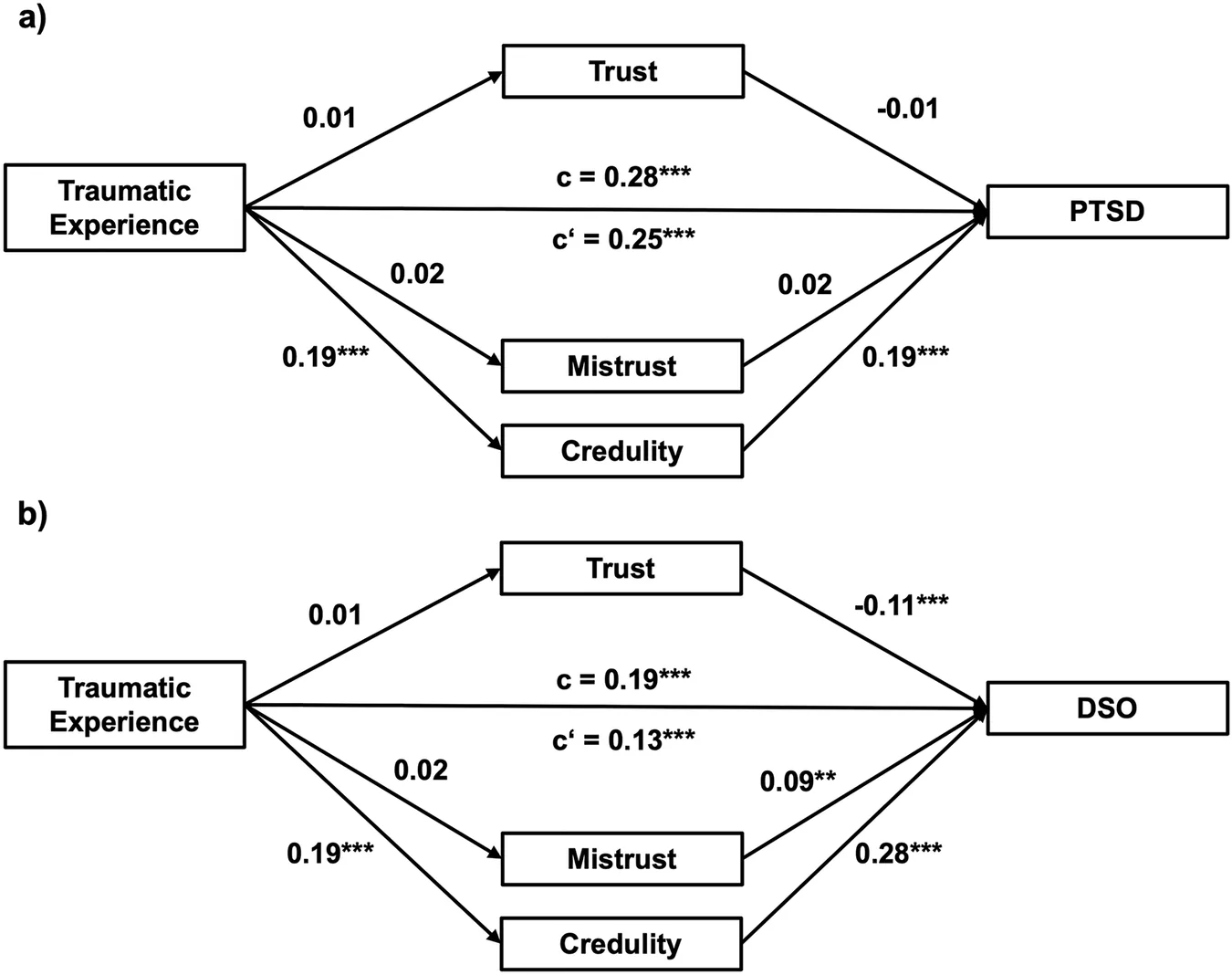
Mediation of traumatic experiences on PTSD and DSO across epistemic stance. The International Trauma Questionnaire [54] was used to assess symptoms of **a** PTSD and **b** disturbances in self-organization (DSO); each panel shows the respective outcome. Epistemic trust, mistrust, and credulity were assessed with the ETMCQ [11]. The graphs are based on *n* = 1 535 participants. * *p* < 0.05; *** *p* < 0.001.

To test our hypothesis that epistemic stance mediates the relationship between trauma and PTSD symptoms, two mediation analyses were performed. For PTSD symptoms (*R²* = 0.12; *F*[4,1530] = 52.03, *p* < 0.001), credulity emerged as a partial mediator between reported traumatic experience and symptom severity (β = 0.04; 95% CI [0.02, 0.05]); epistemic trust (β = 0.00; 95% CI [0.00, 0.00]) and mistrust (β = 0.00; 95% CI [0.00, 0.00]) showed no significant indirect effects (Fig. 2a).

For the cPTSD scale, DSO (*R*^*2*^ = 0.17; *F*[4,1530] = 80.91, *p* < 0.001), we again found credulity (β = 0.05; 95% CI [0.04, 0.07]) but not trust (β = 0.00; 95% CI [-0.01, 0.01]) or mistrust (β = 0.00; 95% CI [0.00, 0.01]) to indirectly link trauma exposure with symptom load (Fig. 2b). All parameters of the two mediation analyses are fully detailed in Supplementary Table 6.

In summary, credulity consistently emerged as a significant mediator linking traumatic experiences to PTSD and cPTSD symptomatology. Although indirect effects were modest, they persisted after controlling for covariates (Supplementary Table 7).

### Full conceptual model

**Fig. 3:**
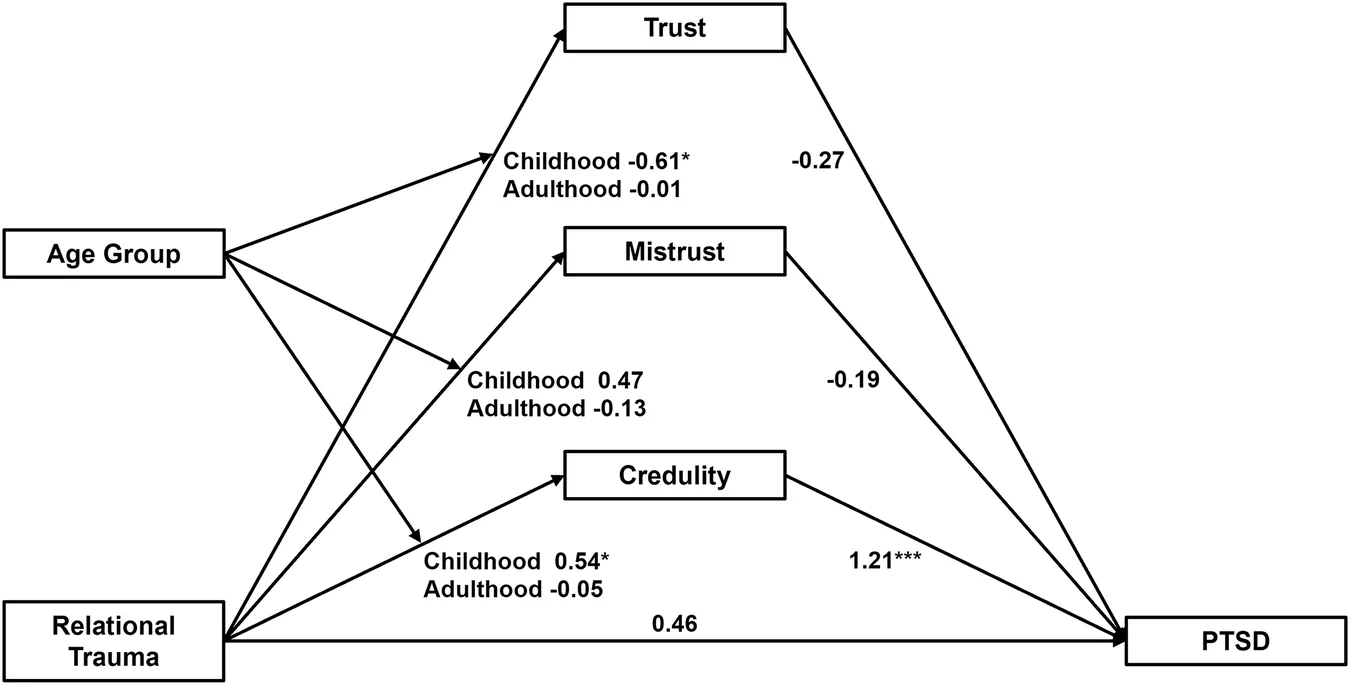
Moderated mediation of relational trauma on PTSD symptoms. The International Trauma Questionnaire [54] was used to assess the presence or absence of relational trauma, as well as symptoms of PTSD. Childhood refers to ages ≤ 18 years, Adulthood to ages > 18 years. Epistemic trust, mistrust, and credulity were assessed with the ETMCQ [11]. The model was conducted with *n* = 838 participants. Coefficients are standardized. * *p* < 0.05; *** *p* < 0.001.

Testing the full conceptual model, we examined how relational trauma predicts epistemic stance depending on age at trauma, subsequently relating to PTSD and DSO symptoms. Figure 3 illustrates the structure of the moderated mediation model for PTSD symptoms. Conditional effects of age at trauma on epistemic stance, as well as conditional indirect effects on the respective outcomes (PTSD and DSO) are summarized in Table 4, with full parameters of all regression paths reported in Supplementary Table 8. The key findings relevant to the hypotheses are outlined in the following for each model.

**Table 4:** Conditional effects of relational trauma on epistemic stance and PTSD symptoms by age at trauma.

	PTSD				DSO			
	β	95% CI	*t*	*p*	β	95% CI	*t*	*p*
DV: Epistemic Stance								
Trust								
Childhood	−0.61	−1.09, −0.13	−2.48	0.014	−0.63	−1.10, −0.15	−2.57	0.010
Adulthood	0.00	−0.16, 0.14	−0.12	0.903	−0.01	−0.16, 0.14	−0.18	0.861
Mistrust								
Childhood	0.47	−0.05, 0.99	1.79	0.074	0.47	−0.04, 0.98	1.80	0.072
Adulthood	−0.13	−0.29, 0.03	−1.55	0.122	−0.12	−0.28, 0.04	−1.47	0.141
Credulity								
Childhood	0.54	0.04, 1.04	2.14	0.033	0.54	0.04, 1.03	2.12	0.034
Adulthood	−0.05	−0.21, 0.11	−0.64	0.524	−0.04	−0.19, 0.12	−0.47	0.638
DV: PTSD/ DSO	(*R²* = 0.27)				(*R²* = 0.43)			
Relational Trauma	0.46	−0.16, 1.09			0.26	−0.27, 0.79		
Trust								
Childhood	0.16	−0.03, 0.46			0.32	0.05, 0.66		
Adulthood	0.00	−0.05, 0.06			0.01	−0.07, 0.09		
Mistrust								
Childhood	−0.09	−0.37, 0.07			0.20	−0.07, 0.59		
Adulthood	0.02	−0.02, 0.09			−0.05	−0.15, 0.01		
Credulity								
Childhood	0.66	0.06, 1.32			0.67	0.01, 1.35		
Adulthood	−0.06	−0.27, 0.13			−0.05	0.25, 0.14		

Conditional effects are presented for two moderated mediation models predicting PTSD and DSO, respectively. The first set of parameters reflects the effects of relational trauma (occurring in childhood vs. adulthood) on the three epistemic stance dimensions (epistemic trust, mistrust, and credulity), which serve as dependent variables (DV). The second set of parameters reports the direct and conditional indirect effects of relational trauma (childhood vs. adulthood) on PTSD or DSO (DV) via epistemic stance. Epistemic trust, mistrust, and credulity were assessed with the ETMCQ [11]. Childhood refers to ages ≤ 18 years, adulthood to ages > 18 years. The International Trauma Questionnaire [54] was used to assess symptoms of PTSD and disturbances in self-organization (DSO). Models were conducted with *n* = 838 (PTSD) and *n* = 848 (DSO) participants. Indirect effects (β) and 95% confidence intervals (CI) were obtained using Model 7 in PROCESS [64] with 5 000 bootstrapped resamples.

#### Effects on PTSD

Conditional effect analyses (Table 4) indicated that relational trauma occurring during childhood was associated with decreased epistemic trust and increased credulity. This effect was not significant for mistrust, nor were there significant effects on epistemic stance from relational trauma experienced during adulthood.

Although the direct effect of relational compared to non-relational trauma on PTSD symptoms was not significant, a significant indirect effect emerged through credulity when relational trauma occurred in childhood. The significant index of moderated mediation (IMM = -0.72, 95% CI [-1.44, -0.08]) indicated that the detrimental indirect effect of relational trauma on PTSD via increased credulity was stronger for earlier compared to later trauma exposure.

These findings suggest that age at trauma moderates the indirect effect of relational trauma on PTSD symptoms through credulity.

#### Effects on DSO

Similarly, relational trauma during childhood – but not adulthood – was significantly associated with decreased epistemic trust and increased credulity. Again, no significant effects were found for mistrust.

As with PTSD, relational trauma had no significant direct effect on DSO symptoms. However, significant indirect effects through epistemic trust and credulity emerged for childhood relational trauma. Both moderated mediation indices were significant (trust: IMM = -0.31, 95% CI [-0.65, -0.03]; credulity: IMM = -0.71, 95% CI [-1.42, -0.05]), indicating that relational trauma is associated with more DSO symptoms via disruptions in epistemic stance, specifically when occurring earlier in life.

Summarizing the conceptual model analyses, relational trauma was associated with disruptions in epistemic stance – particularly reduced trust and increased credulity – only when experienced during childhood. Such epistemic disruptions were, in turn, associated with elevated PTSD and DSO symptom severity.

### Exploratory analysis: sequential moderated mediation model

In a final exploratory step, we tested a sequential moderated mediation model exploring whether early relational trauma and subsequent epistemic disruptions contributed to impaired self-organization, thus exacerbating PTSD symptoms. Results supported this sequential indirect pathway (fully detailed in Supplementary Note 3 and Supplementary Figure 1): relational trauma in childhood predicted reduced epistemic trust and increased credulity, associated with greater DSO symptoms, which were further associated with heightened PTSD symptoms. Neither adulthood trauma nor the mistrust dimension demonstrated significant effects in this model.

## Discussion

This study aimed to extend empirical support for the epistemic trust framework of developmental psychopathology. To this end, we examined the effects of trauma timing and type using a comprehensive analytic approach that accounted for a broad range of traumatic experiences across age groups. Drawing on data from a large representative German sample, we explored how specific trauma characteristics relate to disrupted epistemic stance and, in turn, to PTSD and cPTSD symptoms.

Our findings revealed no robust main effects for trauma timing or type alone. However, a significant interaction emerged, supporting the hypothesis that early relational trauma uniquely predicts disruptions of epistemic stance. Specifically, epistemic credulity emerged as a key mediator, linking relational trauma experienced during childhood – but not adulthood – to elevated PTSD and cPTSD symptoms. Although requiring replication, these results suggest developmental specificity in trauma-related epistemic disruptions, nuancing conceptualizations of how relational trauma contributes to psychopathology.

The finding that trauma is associated with disrupted epistemic stance, particularly elevated credulity, aligns with prior cross-sectional research [11, 20, 21, 32]. While previous studies primarily emphasized childhood adversity, we expanded this perspective by including adult trauma. We found that both childhood and adulthood trauma were associated with elevated credulity relative to non-traumatized individuals. Contrary to our first hypothesis, the strongest contrasts emerged for traumatic events during younger, middle, and older adulthood. This was not explained by trauma recency, which was tested exploratively, nor by participants’ current age, as controlling for age at participation did not alter the results in any of the models.

However, our trauma measure (ITQ [55]) captured only the most salient trauma, potentially neglecting earlier experiences that profoundly shape epistemic stance. Given the cumulative nature of trauma and heightened re-traumatization risk following childhood adversity [43, 44], early adverse experiences likely continue to influence epistemic stance beyond the reported event. Epistemic stance develops early alongside other forms of social cognition [13, 19, 29, 30], suggesting sustained vulnerability. Analogously, research on depression indicates that early independent stressors (e.g., abuse and neglect) increase the risk of subsequent dependent stressors (e.g., maladaptive relationship patterns), perpetuating cycles of interpersonal disruption and psychopathology [66–68]. This recursive vulnerability may also extend to epistemic disruption, potentially contributing to its persistence even when only salient traumatic events are measured. Consequently, inconsistent findings regarding trauma timing [46, 47] may stem not only from recall or measurement limitations but also from the inherent biopsychosocial complexity underlying trauma responses [69].

A similar pattern emerged regarding trauma type. Contrary to our second hypothesis, the overall distinction between relational and non-relational trauma did not yield consistent differences in epistemic stance, although certain experiences – particularly sexualized violence – were significantly associated with elevated credulity. This contrasts with prior research linking interpersonal violence to impairments in personality functioning, mentalization, and attachment [27, 70–73] – constructs closely related to epistemic trust [11, 13, 20]. Using an exploratory coding approach based on actively inflicted harm clarified that intentional trauma was associated with higher credulity than non-intentional trauma or no trauma. This aligns with previous studies identifying assaultive interpersonal trauma as especially detrimental [41, 44, 51, 52] and suggests that non-intentional relational trauma, such as bereavement, may have a comparatively weaker impact on epistemic trust.

Our difficulty in identifying robust main effects for individual trauma characteristics is consistent with prior research. Although trauma timing and type are frequently explored predictors of PTSD, meta-analyses indicate that they typically explain only modest variance in symptoms [43, 69]. Individual responses to trauma – shaped by subjective appraisal, emotional processing, and pre-existing vulnerabilities – may play a more decisive role [74–76]. Such complexity could explain why our findings did not support straightforward main effects of trauma timing or type and instead highlight the importance of their interaction within a developmental framework.

Indeed, when combining trauma characteristics, results aligned well with our conceptual model. Relational trauma in childhood, but not adulthood, was significantly associated with disruptions across epistemic trust, mistrust, and credulity. Interestingly, intentional trauma predicted greater credulity regardless of timing, suggesting that intentional interpersonal harm exerts detrimental effects on epistemic stance independent of age at trauma. Comparable age-insensitive effects have been documented for related outcomes, including attachment insecurity and cPTSD [42, 77].

Last, our third hypothesis that disrupted epistemic stance mediates the association between lifetime trauma and (c)PTSD symptoms received partial support. Previous research has reported mediation effects of epistemic stance in the association between childhood adversity and PTSD [11, 21, 32]. Extending this, we found that epistemic credulity accounted for small yet consistent indirect associations between trauma exposure and increased PTSD and DSO symptom severity across age and trauma types. In contrast, neither epistemic trust nor mistrust demonstrated significant indirect effects in these relationships. These results partially replicate earlier findings associating epistemic credulity (and mistrust) with various psychopathological outcomes, including PTSD [11, 20, 21, 32, 37].

When trauma timing and type were jointly considered, explained variance more than doubled – rising substantially from 12% to 27% for PTSD, and from 17% to 43% for DSO. Supporting our hypotheses, relational trauma during childhood, but not adulthood, was significantly associated with disrupted epistemic trust and heightened credulity, which in turn predicted elevated PTSD and DSO symptoms. These findings align with developmental accounts of epistemic trust as a foundational socio-cognitive mechanism shaped by early relational experiences [18, 19, 28–30]. Similar developmental pathways have been demonstrated for related constructs, including mentalizing, attachment, and personality functioning, linking trauma-induced disruptions to diverse psychopathological outcomes beyond PTSD and cPTSD, such as depression, anxiety, and somatic health difficulties [23–27]. To our knowledge, this is the first study to explicitly contrast childhood and adulthood trauma within the epistemic trust framework, providing preliminary evidence for the unique role of early relational trauma in shaping epistemic stance and, consequently, foundational capacities for social learning and relationship formation. Exploratory sequential mediation analyses, together with previous research [32], suggest that early disruptions in epistemic trust may contribute to broader personality disturbances (e.g., self-organization, personality functioning) and increased vulnerability to subsequent adversity. However, given their exploratory and cross-sectional nature, these analyses do not permit causal interpretation and warrant replication in future longitudinal studies.

Notably, epistemic credulity emerged as the epistemic stance dimension most sensitive to trauma across analyses, whereas trust and mistrust effects were less robust. This pattern aligns with prior work suggesting that credulity may constitute a particularly consequential interpersonal vulnerability. Elevated credulity has been linked to lower self-efficacy and agency, ineffective mentalizing, and emotion dysregulation – established vulnerability factors for psychopathology [11, 21, 78]. In line, several studies have reported that credulity shows particularly strong associations with personality dysfunction, depression, anxiety, and PTSD symptoms, and often functions as a more robust mediator of trauma effects than mistrust or trust [11, 21, 32, 78]. In the present study, the observed indirect effects via credulity were small in magnitude, but consistent across models, suggesting a modest yet potentially meaningful contribution within a broader network of risk factors. Credulity has further been linked to treatment-related change in PTSD and DSO severity, showing the strongest outcome associations [79]. Theoretically, heightened credulity may increase vulnerability to revictimization – a known risk among trauma survivors [44] – by compromising risk perception and facilitating susceptibility to manipulation, coercion, or interpersonal exploitation [11]. Further, the diminished sense of agency and ultimately exhaustion of the epistemic system associated with credulity may hinder the capacity to benefit from supportive or “corrective” relationships [11, 20].

At the same time, methodological explanations remain plausible and warrant cautious interpretation. The version of the ETMCQ used in this study has shown suboptimal psychometric performance of the mistrust scale, potentially exaggerating the relative prominence of credulity. A revised measure with improved psychometric properties (ETMCQ-R [22]) is now available and may help clarify these distinctions in future work. Moreover, some studies highlight mistrust, rather than credulity, as the most predictive dimension, including findings of stronger mistrust-psychopathology associations in non-clinical samples [20] and evidence that mistrust can be a central mediator of clinical change [36]. These mixed results suggest that the relative importance of credulity and mistrust may depend on the questionnaire version employed and may reflect differential relevance across contexts (e.g., clinical versus non-clinical) and outcomes (e.g., vulnerability versus treatment processes).

Overall, our findings strengthen emerging evidence that epistemic credulity may represent one potential pathway linking trauma to psychosocial dysfunction, while also underscoring the need for longitudinal and clinical studies to determine to what extent its salience reflects a trauma-induced mechanism, a preexisting vulnerability, or a methodological artifact.

Integrating our findings with existing literature, epistemic trust, mistrust, and credulity – constructs that have received increasing attention over the past decade – appear to play a relevant role for understanding mental health and psychopathology. Epistemic trust is crucial for successful psychotherapy, facilitating social learning and therapeutic benefit [12, 28, 80]. This study focused on epistemic stance in the context of trauma and (c)PTSD, conditions often marked by treatment resistance, dropout, and persistent symptoms [37, 81, 82]. Identifying epistemic stance as a mechanism linking trauma to psychopathology points toward restoring epistemic trust as a promising therapeutic target – an area just beginning to be explored. Initial longitudinal evidence links improvements in epistemic stance with reductions in trauma-related symptoms [36, 83]. For instance, Lampe and colleagues [79] recently demonstrated that increased epistemic trust and decreased credulity during psychodynamic inpatient treatment predicted symptom improvement in trauma-exposed patients.

### Limitations and directions for future research

A primary limitation of this study is its cross-sectional design, precluding causal interpretations of the examined pathways. In particular, the temporal sequence of trauma exposure, epistemic stance disruptions, and PTSD/DSO symptoms cannot be established, and bidirectional relationships between these constructs cannot be ruled out. Given the developmental orientation of our conceptual framework, future studies employing longitudinal designs are needed to capture temporal dynamics and developmental trajectories of epistemic trust disruptions following trauma.

While employing a large representative community sample strengthens generalizability, disrupted epistemic stance is likely even more pronounced in clinical populations. Future investigations should extend the evidence to high-risk or clinical samples. Moreover, as information on participants’ race and ethnicity was not available, the role of racial and ethnic diversity could not be examined, which limits the generalizability of the findings.

Another limitation concerns the suboptimal internal consistency of the ETMCQ mistrust scale in our sample. It aligns with prior studies reporting psychometric instability of this subscale in community samples [11, 78], and may partly explain the less consistent mistrust effects observed in our analyses. Recent work has identified similar issues, leading to the development of the revised ETMCQ-R [22], which improves the psychometric properties of the mistrust and trust subscales. Using this updated measure in future research may help to clarify the relative roles of mistrust and credulity as distinct, context-dependent strategies that differentially shape vulnerability and treatment response in trauma-related psychopathology.

A further challenge lies in the use of retrospective self-report for trauma assessment. Because the ITQ requires respondents to report only their most distressing event, our data capture a single, self-selected trauma per participant. This limits the precision of our age-group classifications and may partly account for the absence of age-group effects on epistemic stance for several reasons. First, this design cannot distinguish between individuals with isolated versus multiple traumatic experiences, precluding the examination of cumulative or developmental trauma trajectories. Second, recency effects and other recall biases may influence which event participants select when they have experienced multiple relevant incidents across the life span [84] – possibly explaining why only a relatively low proportion of the indexed traumatic events occurred during childhood (*n* = 106). Third, prior research shows low concordance between self-reported and documented trauma [85]. Our findings suggest that individuals with lower epistemic trust may be less likely to disclose trauma, indicating that reporting is shaped not only by memory but also by interpersonal openness. Future research should therefore employ multi-event, developmentally sensitive trauma assessments that allow for mapping of trajectories, chronicity, and accumulation of adverse experiences.

A final and key limitation concerns the constraints of our trauma type categorization. The open-text trauma descriptions available in the dataset provided limited detail, necessitating a pragmatic coding approach. Although categorizations were grounded in DSM-5 definitions and broadened to include relational experiences relevant to DSO, some interpretive decisions were unavoidable, particularly when severity, exposure type, or relational context were unclear. The lack of detail limits the accuracy with which trauma characteristics could be examined. Future studies would benefit from more detailed, structured trauma assessments that capture event features, context, and subjective impact more comprehensively.

## Conclusion

This study provides novel empirical evidence consistent with a developmental model of epistemic trust, suggesting that early relational trauma is associated with disruptions in adaptive trust in interpersonal communication, thereby contributing to trauma-related psychopathology. By systematically examining trauma timing and type, our findings indicate that relational context during sensitive developmental periods, rather than trauma exposure alone, may critically shape epistemic stance disruptions. Epistemic credulity, in particular, emerged as a potential mechanism linking trauma to PTSD and cPTSD symptoms. These insights advance our understanding of how trauma may undermine foundational socio-cognitive processes and offer further indications for targeting epistemic trust in psychotherapeutic intervention efforts.

## Supplementary information

Supplemental Material

## Data Availability

Analysis scripts along with raw data supporting the conclusions of this study are publicly accessible at https://osf.io/kfrez/.

## Open science and transparency

Hypotheses and methods of this study were preregistered at https://osf.io/cxvej. The materials used in this study are widely available.
