# Supplementary material for: Cracks in the foundation: disrupted epistemic trust as a link between trauma, PTSD and disturbances in self-organization
Source: Transl Psychiatry. 2026 Sep 17;16:471. doi: 10.1038/s41398-026-04442-3 (PMC13586315; doi:10.1038/s41398-026-04442-3)
Supplement: Supplementary file 1 [file 41398_2026_4442_MOESM1_ESM.docx]

**Supplementary Table 1**

*Coding Scheme for Trauma Characterization*

|  | Description | Code | Notes |
| --- | --- | --- | --- |
| Presence of trauma |  |  |  |
| yes | Traumatic event described | 1 | Cases with inconclusive responses or missing information were manually reviewed and recoded as needed.  Text answers explicitly denying trauma (e.g., “none,” “no,” “never”) were recoded as 0 (no trauma). |
| no | No traumatic event described | 0 |  |
| not specified | Response missing or unclear | 99 |  |
| Age at trauma |  |  |  |
| childhood | ≤ 18 years | 1 | Age at trauma was inferred from participants’ current age and the reported recency of the event. Trauma reported by underaged participants (*n* = 43) automatically fell into the “childhood” category.  All available contextual information (e.g., trauma description, reference to historical events) was used for manual classification.  If the range was too broad to determine a single age group and no further information was available, the case was coded as “not specified.” |
| adulthood | > 18 years | 2 |  |
| not specified | Response missing or age range too broad | NA |  |
| Age subgroup at trauma |  |  |  |
| childhood | ≤ 12 years | 1 | Age at trauma was inferred from participants’ current age and the reported recency of the event.  All available contextual information (e.g., trauma description, reference to historical events) was used for manual classification.  If the range was too broad to determine a single age subgroup and no further information was available, the case was coded as “not specified.” |
| adolescence | 13 – 18 years | 2 |  |
| young adulthood | 19 – 40 years | 3 |  |
| middle adulthood | 41 – 65 years | 4 |  |
| older adulthood | > 65 years | 5 |  |
| not specified | Response missing or age range too broad | NA |  |
| Type of trauma |  |  |  |
| loss | Death of a close person | 1 | Cases where accidents or illness resulted in the death of a close person were coded as 1 (“loss”).  Events that were witnessed or learned about (e.g., a serious accident, a partner’s or parent’s severe illness) were coded according to the corresponding trauma category. |
| separation | Non-fatal loss of a significant relationship (e.g., abandonment, divorce) | 2 |  |
| emotional violence | Psychological abuse, neglect, humiliation | 3 |  |
| physical violence | Physical assault, domestic violence | 4 |  |
| sexualized violence | Sexual abuse or assault | 5 |  |
| war/ terror | Exposure to combat, terrorism, displacement | 6 |  |
| natural disaster | E.g. earthquake, flood, fire, etc. | 7 |  |
| accident | E.g. traffic or workplace accidents | 8 |  |
| illness/ surgery | Severe physical or mental illness, major surgery | 9 |  |
| other | Events not fitting other categories (e.g., COVID-19 pandemic, economic hardship, imprisonment, death of pets) | 10 |  |
| not specified | Response missing or unclear | NA |  |
| Nature of trauma |  |  |  |
| relational | Interpersonal harm or relationship ruptures | 1 | Relational trauma included: Loss, separation, emotional violence, physical violence, sexualized violence |
| non-relational | Not primarily interpersonal in nature | 0 | Non-relational trauma included: War/terror, natural disaster, accident, illness/surgery |
| intentional | Directed purposeful harm by another person | 1 | Intentional trauma included: Emotional violence, physical violence, sexualized violence |
| non-intentional | Accidental or situational trauma | 0 | Non-intentional trauma included: Loss, separation, war/terror, natural disaster, accident, illness/surgery |
| not specified | Trauma type not specified | NA |  |

**Supplementary Table 2**

*Group Differences Between Participants With and Without a Traumatic Experience*

|  | Presence of trauma | | | *F* | *p* | η^2^ |
| --- | --- | --- | --- | --- | --- | --- |
|  | yes | no | not disclosed |  |  |  |
| Epistemic Stance |  |  |  | 20.50 | < .001 | 0.03 |
| Trust | 25.4 (5.0) | 25.4 (4.9) | 24.3 (5.1) | 11.52 | < .001 | 0.01 |
| Mistrust | 11.1 (3.8) | 11.0 (3.5) | 10.6 (3.3) | 4.41 | .012 | 0.00 |
| Credulity | 12.6 (5.1) | 10.5 (5.0) | 11.7 (4.7) | 30.89 | < .001 | 0.03 |
| PTSD | 10.0 (4.4) | 6.3 (1.4) | 6.6 (1.9) | 197.50 | < .001 | 0.19 |
| DSO | 9.4 (4.1) | 7.4 (2.5) | 8.1 (3.2) | 38.14 | < .001 | 0.04 |
| ACE | 1.2 (1.9) | 0.4 (1.2) | 0.5 (1.3) | 37.34 | < .001 | 0.04 |

*Note.* Descriptive values follow the structure *M* (*SD*). MANOVA results for the epistemic stance and univariate ANOVAS for each ETMCQ^11^ subscale, i.e., trust, mistrust, and credulity are reported. Symptoms of PTSD and disturbances in self-organization (DSO) were assessed with the ITQ^55^. Adverse childhood experiences were measured with the ACE^61^. Models were conducted with *n* = 2 235 (ETMCQ) and *n* = 1 738 (other scales) participants.

**Supplementary Table 3**

*Construct, Criterion, and Convergent Validity of Trauma Coding Controlling for Covariates*

|  | Trust | | | Mistrust | | | Credulity | | |
| --- | --- | --- | --- | --- | --- | --- | --- | --- | --- |
|  | *F* | *p* | η_p_^2^ | *F* | *p* | η_p_^2^ | *F* | *p* | η_p_^2^ |
| Trauma | 12.71 | < .001 | 0.01 | 4.18 | .015 | 0.00 | 31.53 | < .001 | 0.03 |
| Age | 1.41 | .235 | 0.00 | 6.54 | .011 | 0.00 | 1.03 | .310 | 0.00 |
| Sex | 16.84 | < .001 | 0.02 | 0.31 | .737 | 0.00 | 9.65 | < .001 | 0.00 |
| Education | 0.11 | .745 | 0.00 | 3.55 | .060 | 0.00 | 62.70 | < .001 | 0.03 |
| Income | 4.07 | .017 | 0.00 | 9.40 | < .001 | 0.00 | 6.86 | .001 | 0.00 |
|  |  |  |  |  |  |  |  |  |  |
|  | PTSD | | | DSO | | | ACE | | |
|  | *F* | *p* | η_p_^2^ | *F* | *p* | η_p_^2^ | *F* | *p* | η_p_^2^ |
| Trauma | 197.69 | < .001 | 0.19 | 38.05 | < .001 | 0.04 | 37.89 | < .001 | 0.04 |
| Age | 3.84 | .050 | 0.00 | 2.15 | .143 | 0.00 | 2.97 | .085 | 0.00 |
| Sex | 5.13 | .006 | 0.00 | 0.30 | .739 | 0.00 | 0.15 | .862 | 0.00 |
| Education | 3.61 | .058 | 0.00 | 8.29 | .004 | 0.00 | 7.59 | .006 | 0.00 |
| Income | 3.05 | .048 | 0.00 | 22.97 | < .001 | 0.02 | 20.90 | < .001 | 0.02 |

*Note.* Test statistics were obtained using a MANCOVA for the epistemic stance (*F*[6,4416] = 21.39, *p* < .001, η_p_^2^ = 0.03) along with univariate ANCOVAs. The ETMCQ^11^ was used to measure epistemic trust, mistrust, and credulity. The ITQ^55^ was used to assess symptoms of PTSD and disturbances in self-organization (DSO). Adverse childhood experiences were measured with the ACE^61^. Models were conducted with *n* = 2 218 (ETMCQ) and *n* = 1 723 (other scales) participants.

**Supplementary Note 1**

***Validity Check***

Epistemic stance differed between people who indicated a traumatic event, the absence of traumatic events, or chose not to make a statement. There was no difference between participants reporting the presence or absence of trauma in epistemic trust (*d* = 0; 95% CI [-0.11, 0.12]; *p* = .996) or mistrust (*d* = 0.02; 95% CI [-0.09, 0.13]; *p* = .934). Interestingly, it was those who chose not to disclose that demonstrated lower epistemic trust than participants who reported trauma (*d* = -0.22; 95% CI [-0.31, -0.12]; *p* < .001) or no trauma (*d* = -0.21; 95% CI [-0.33, -0.1]; *p* = .001). They also had lower mistrust scores than those with trauma (*d* = -0.14; 95% CI [-0.23, -0.04]; *p* = .011) but not than those without (*d* = -0.12; 95% CI [-0.24, -0.01]; *p* = .118). For credulity, people with trauma exhibited significantly higher credulity than people without trauma (*d* = 0.43; 95% CI [0.31, 0.54]; *p* < .001) and those who did not disclose (*d* = 0.2; 95% CI [0.1, 0.29]; *p* < .001). Compared to participants who reported no trauma, those who did not specify had higher credulity scores (*d* = 0.25; 95% CI [0.13, 0.36]; *p* < .001). In summary, the results of our construct check show that a traumatic event may be associated with disruptions in epistemic stance, especially credulity. Additionally, we found indications that the self-report of traumatic events may be biased by the epistemic stance.

The validity checks for our coding scheme yielded criterion validity with the ITQ. PTSD symptoms were stronger in participants with a history of trauma compared to those with no trauma (*d* = 0.91; 95% CI [0.76, 1.06]; *p* < .001) or those who did not disclose (*d* = 0.89; 95% CI [0.78, 1]; *p* < .001). There was no difference between people without trauma and those who did not specify (*d* = 0.16; 95% CI [0, 0.32]; *p* = .597). The same pattern emerged for DSO, with stronger symptoms for those with trauma than those without (*d* = 0.51; 95% CI [0.37, 0.66]; *p* < .001) or those who did not specify (*d* = 0.34; 95% CI [0.23, 0.45]; *p* < .001). People without trauma tended to display lower levels of DSO than those who did not disclose (*d* = -0.22; 95% CI [-0.38, -0.07]; *p* = .061), but this difference did not reach statistical significance.

Convergent validity with the ACE was established, showing that participants who indicated trauma on the ITQ also reported more traumatic events during childhood than participants who indicated no trauma (*d* = 0.43; 95% CI [0.28, 0.57]; *p* < .001) or did not disclose (*d* = 0.39; 95% CI [0.28, 0.5]; *p* < .001). Again, no difference was found between those who did not indicate trauma and those who did not specify (*d* = 0.07; 95% CI [-0.09, 0.22]; *p* = .805).

Analyses controlling for age, sex, education, and income yield the same result pattern for all models. In detail, trust was negatively associated with male gender and positively with income. Mistrust positively related to age and negatively to income. Credulity decreased with male gender, education, and income. PTSD symptoms were negatively correlated with age, male gender, and income. Both DSO and ACE scores showed negative associations with education and income.

Taken together, our construct check indicates effects of our trauma presence coding on our key epistemic stance variables and demonstrates criterion and convergent validity of our coding scheme.

**Supplementary Table 4**

*MANCOVA Results for Group Differences in Epistemic Stance Between Age and Type Groups*

|  | Trust | | |  | Mistrust | | |  | Credulity | | |  | MANCOVA | | |
| --- | --- | --- | --- | --- | --- | --- | --- | --- | --- | --- | --- | --- | --- | --- | --- |
|  | *F* | *p* | η_p_^2^ |  | *F* | *p* | η_p_^2^ |  | *F* | *p* | η_p_^2^ |  | *F* | *p* | η_p_^2^ |
| Age at trauma | 0.64 | .528 | 0.00 |  | 0.17 | .845 | 0.00 |  | 27.00 | < .001 | 0.04 |  | 13.33 | < .001 | 0.03 |
| Current age | 0.48 | .489 | 0.00 |  | 3.06 | .081 | 0.00 |  | 4.74 | .030 | 0.00 |  | 6.51 | < .001 | 0.01 |
| Gender | 6.32 | .002 | 0.01 |  | 1.29 | .276 | 0.00 |  | 6.35 | .002 | 0.01 |  | 5.27 | < .001 | 0.01 |
| Education | 0.19 | .666 | 0.00 |  | 0.10 | .746 | 0.00 |  | 30.54 | < .001 | 0.02 |  | 14.79 | < .001 | 0.03 |
| Income | 2.26 | .105 | 0.00 |  | 3.28 | .038 | 0.00 |  | 2.37 | .093 | 0.00 |  | 1.72 | .112 | 0.00 |
|  |  |  |  |  |  |  |  |  |  |  |  |  |  |  |  |
| Age subgroup at trauma | 0.59 | .706 | 0.00 |  | 0.92 | .466 | 0.00 |  | 12.64 | < .001 | 0.05 |  | 6.26 | < .001 | 0.03 |
| Current age | 0.03 | .853 | 0.00 |  | 9.95 | .002 | 0.01 |  | 0.00 | .968 | 0.00 |  | 5.19 | .001 | 0.01 |
| Gender | 5.18 | .006 | 0.01 |  | 0.68 | .508 | 0.00 |  | 5.42 | .005 | 0.01 |  | 4.57 | < .001 | 0.01 |
| Education | 0.09 | .769 | 0.00 |  | 0.50 | .479 | 0.00 |  | 36.21 | < .001 | 0.03 |  | 16.18 | < .001 | 0.04 |
| Income | 1.85 | .158 | 0.00 |  | 4.20 | .015 | 0.01 |  | 2.83 | .060 | 0.00 |  | 1.99 | .064 | 0.01 |
|  |  |  |  |  |  |  |  |  |  |  |  |  |  |  |  |
| Trauma nature | 0.87 | .421 | 0.00 |  | 0.61 | .546 | 0.00 |  | 25.38 | < .001 | 0.03 |  | 13.56 | < .001 | 0.03 |
| Current age | 0.48 | .488 | 0.00 |  | 4.90 | .027 | 0.00 |  | 1.73 | .189 | 0.00 |  | 5.21 | .001 | 0.01 |
| Gender | 8.73 | < .001 | 0.01 |  | 0.73 | .480 | 0.00 |  | 9.75 | < .001 | 0.01 |  | 7.88 | < .001 | 0.02 |
| Education | 0.49 | .483 | 0.00 |  | 0.62 | .433 | 0.00 |  | 34.55 | < .001 | 0.02 |  | 15.26 | < .001 | 0.03 |
| Income | 2.96 | .052 | 0.00 |  | 5.54 | .004 | 0.01 |  | 2.90 | .056 | 0.00 |  | 2.44 | .024 | 0.01 |
|  |  |  |  |  |  |  |  |  |  |  |  |  |  |  |  |
| Trauma type | 1.40 | .182 | 0.01 |  | 1.32 | .224 | 0.01 |  | 7.54 | < .001 | 0.05 |  | 4.21 | < .001 | 0.03 |
| Current age | 0.24 | .627 | 0.00 |  | 7.13 | .008 | 0.01 |  | 0.45 | .501 | 0.00 |  | 4.96 | .002 | 0.01 |
| Gender | 8.19 | < .001 | 0.01 |  | 0.59 | .555 | 0.00 |  | 8.69 | < .001 | 0.01 |  | 7.34 | < .001 | 0.02 |
| Education | 0.57 | .450 | 0.00 |  | 0.66 | .415 | 0.00 |  | 34.85 | < .001 | 0.02 |  | 15.26 | < .001 | 0.03 |
| Income | 3.21 | .040 | 0.00 |  | 5.26 | .005 | 0.01 |  | 2.51 | .082 | 0.00 |  | 2.40 | .026 | 0.00 |

*Note.* MANCOVAs and univariate ANCOVAS for each ETMCQ^11^ subscale, i.e., trust, mistrust, and credulity, are reported. Results are reported for the grouping factors of interest (age or age subgroup at trauma and trauma nature or type) as well as all covariates. Models were conducted with *n* = 1 330 (age group), *n* = 1 186 (age subgroup), and *n* = 1 423 (trauma nature and type) participants.

**Supplementary Note 2**

***H2: Type of Trauma Using an Alternative Coding Scheme***

An exploratory MANOVA compared the effects of trauma that was intentionally inflicted on an individual by another person (i.e., emotional, physical, and sexualized violence; *n* = 82; 3.3%) with non-intentional trauma (i.e., loss, separation, war and terror, natural disaster, accident, illness and surgery; *n* = 932; 37.0%) and no trauma (*n* = 465; 30.7%).

Like for relational versus non-relational trauma, univariate differences in credulity were observed: while both intentional (*d* = 0.75; 95% CI [0.51, 0.99]; *p* < .001) and non-intentional (*d* = 0.37; 95% CI [0.26, 0.49]; *p* < .001) trauma were again associated with increased credulity compared to no trauma, participants that reported intentional trauma also demonstrated significantly higher credulity than those with other traumas (*d* = 0.36; 95% CI [0.13, 0.59]; *p* = .005). In conclusion, these additional analyses show that it was the intentional rather than relational nature of the trauma, which was associated with increased credulity.

***Exploratory Analysis: Interactive Effects Between Trauma Characteristics* *Using an Alternative Coding Scheme***

We also conducted an exploratory MANOVA testing the interaction between age at trauma and the intentional vs. non-intentional nature of the trauma. This interaction did not reach significance for the multivariate model (*F*[3,846] = 1.88, p = .131), nor for any of the univariate analyses (Trust: *F*[1,848] = 3.77, p = .053; Mistrust: *F*[1,848] = 2.89, p = .089; Credulity: *F*[1,848] = 0.59, p = .444), even if trends for trust and mistrust could be noted. Instead, the main effect of intentionality remained the dominant predictor of epistemic stance (*F*[3,846] = 3.17, p = .024). This indicates that intentional trauma is related to impaired epistemic stance in any age group, whereas the effects of broader relational trauma are more age group specific.

**Supplementary Table 5**

*Group Differences in Epistemic Stance Between Intentional and Non-Intentional Trauma*

|  | Trust | Mistrust | Credulity | MANOVA |
| --- | --- | --- | --- | --- |
| Nature of trauma |  |  |  |  |
| intentional | 25.7 (5.2) | 11.9 (3.9) | 14.2 (5.1)*** |  |
| non-intentional | 25.5 (5.0) | 10.9 (3.4) | 12.4 (5.2)*** |  |
| *no trauma* | *25.4 (4.9)* | *11.0 (3.5)* | *10.5 (5.0)* |  |
|  | *F*(2,1428) = 0.22 | *F*(2,1428) = 2.43 | *F*(2,1428) = 29.65 | *F*(6,2854) = 14.10 |
|  | *p* = .805 | *p* = .088 | *p* < .001 | *p* < .001 |
|  | η_p_^2^ = 0.00 | η_p_^2^ = 0.00 | η_p_^2^ = 0.04 | η_p_^2^ = 0.03 |
|  |  |  |  |  |
|  | Trust | Mistrust | Credulity | MANCOVA |
| Nature of trauma | *F*(2,1414) = 0.21 | *F*(2,1414) = 2.48 | *F*(2,1414) = 30.82 | *F*(6,2826) = 14.79 |
|  | *p* = .808 | *p* = .084 | *p* < .001 | *p* < .001 |
|  | η_p_² = 0.00 | η_p_² = 0.00 | η_p_² = 0.04 | η_p_² = 0.03 |
| Current age | *F*(1,1414) = 0.48 | *F*(1,1414) = 6.45 | *F*(1,1414) = 0.69 | *F*(3,1412) = 4.95 |
|  | *p* = .489 | *p* = .011 | *p* = .405 | *p* = .002 |
|  | η_p_² = 0.00 | η_p_² = 0.00 | η_p_² = 0.00 | η_p_² = 0.01 |
| Gender | *F*(2,1414) = 8.31 | *F*(2,1414) = 0.51 | *F*(2,1414) = 8.94 | *F*(6,2826) = 7.46 |
|  | *p* < .001 | *p* = .600 | *p* < .001 | *p* < .001 |
|  | η_p_² = 0.01 | η_p_² = 0.00 | η_p_² = 0.01 | η_p_² = 0.02 |
| Education | *F*(1,1414) = 0.41 | *F*(1,1414) = 0.64 | *F*(1,1414) = 34.23 | *F*(3,1412) = 15.01 |
|  | *p* = .522 | *p* = .423 | *p* < .001 | *p* < .001 |
|  | η_p_² = 0.00 | η_p_² = 0.00 | η_p_² = 0.02 | η_p_² = 0.03 |
| Income | *F*(2,1414) = 3.06 | *F*(2,1414) = 5.17 | *F*(2,1414) = 2.76 | *F*(6,2826) = 2.35 |
|  | *p* = .047 | *p* = .006 | *p* = .064 | *p* = .029 |
|  | η_p_² = 0.00 | η_p_² = 0.01 | η_p_² = 0.00 | η_p_² = 0.01 |

*Note.* Models were conducted with *n* = 1 431 (no covariates) and *n* = 1 423 (including covariates) participants. Descriptive values follow the structure *M* (*SD*) to represent mean and standard deviation. For each grouping factor MANOVA results including all three ETMCQ^11^ subscales as outcome variables are reported. Univariate ANOVAS for each ETMCQ subscale, i.e., trust, mistrust, and credulity, are reported below the descriptive values. Significant pairwise comparisons between each trauma type and “no trauma”, based on Tukey-Kramer HSD tests, are indicated with asterisks: *** p < .001.

**Supplementary Table 6**

*Mediation Results of Trauma Through Epistemic Stance on PTSD and DSO*

|  | PTSD | | | |  | DSO | | | |
| --- | --- | --- | --- | --- | --- | --- | --- | --- | --- |
|  | β | *SE* | *t* | *p* |  | β | *SE* | *t* | *p* |
| DV: PTSD/ DSO (total effect) |  |  |  |  |  |  |  |  |  |
| Trauma | 0.28 | 0.02 | 11.54 | < .001 |  | 0.19 | 0.03 | 7.50 | < .001 |
| DV: PTSD/ DSO (direct effect) |  |  |  |  |  |  |  |  |  |
| Trauma | 0.25 | 0.02 | 10.03 | < .001 |  | 0.13 | 0.02 | 5.63 | < .001 |
| Trust | -0.01 | 0.02 | -0.52 | .605 |  | -0.11 | 0.02 | -4.83 | < .001 |
| Mistrust | 0.02 | 0.03 | 0.73 | .464 |  | 0.09 | 0.03 | 3.17 | .002 |
| Credulity | 0.19 | 0.03 | 6.21 | < .001 |  | 0.28 | 0.03 | 9.53 | < .001 |
| DV: Trust |  |  |  |  |  |  |  |  |  |
| Trauma | 0.01 | 0.03 | 0.21 | .835 |  | 0.01 | 0.03 | 0.21 | .835 |
| DV: Mistrust |  |  |  |  |  |  |  |  |  |
| Trauma | 0.02 | 0.03 | 0.59 | .555 |  | 0.02 | 0.03 | 0.59 | .555 |
| DV: Credulity |  |  |  |  |  |  |  |  |  |
| Trauma | 0.19 | 0.03 | 7.63 | < .001 |  | 0.19 | 0.03 | 7.63 | < .001 |
|  |  |  |  | |  |  |  |  | |
|  | β | *SE* | 95% CI | |  | β | *SE* | 95% CI | |
| Indirect effects |  |  |  | |  |  |  |  | |
| Total indirect effect | 0.04 | 0.01 | 0.02, 0.05 | |  | 0.05 | 0.01 | 0.03, 0.08 | |
| Trust | 0.00 | 0.00 | 0.00, 0.00 | |  | 0.00 | 0.00 | -0.01, 0.01 | |
| Mistrust | 0.00 | 0.00 | 0.00, 0.00 | |  | 0.00 | 0.00 | 0.00, 0.01 | |
| Credulity | 0.04 | 0.01 | 0.02, 0.05 | |  | 0.05 | 0.01 | 0.04, 0.07 | |

*Note.* All models were conducted with a subsample of *n* = 1 535 participants. Epistemic trust, mistrust, and credulity were assessed with the ETMCQ^11^. The ITQ^55^ was used to assess symptoms of PTSD and disturbances in self-organization (DSO). All coefficients are standardized. Indirect effects were computed with bootstrapping using 5 000 iterations.

**Supplementary Table 7**

*Mediation Results of Trauma Through Epistemic Stance on PTSD and DSO Controlled for Covariates*

|  | PTSD | | | |  | DSO | | | |
| --- | --- | --- | --- | --- | --- | --- | --- | --- | --- |
|  | β | *SE* | *t* | *p* |  | β | *SE* | *t* | *p* |
| DV: PTSD/ DSO (total effect) |  |  |  |  |  |  |  |  |  |
| Trauma | 0.29 | 0.02 | 11.50 | < .001 |  | 0.20 | 0.03 | 7.70 | < .001 |
| DV: PTSD/ DSO (direct effect) |  |  |  |  |  |  |  |  |  |
| Trauma | 0.25 | 0.03 | 10.14 | < .001 |  | 0.15 | 0.02 | 6.02 | < .001 |
| Trust | -0.02 | 0.02 | -0.75 | .452 |  | -0.11 | 0.02 | -4.62 | < .001 |
| Mistrust | 0.03 | 0.03 | 0.90 | .371 |  | 0.09 | 0.03 | 3.26 | .001 |
| Credulity | 0.17 | 0.03 | 5.68 | < .001 |  | 0.27 | 0.03 | 9.02 | < .001 |
| DV: Trust |  |  |  |  |  |  |  |  |  |
| Trauma | 0.00 | 0.03 | -0.04 | .975 |  | 0.00 | 0.03 | -0.04 | .965 |
| DV: Mistrust |  |  |  |  |  |  |  |  |  |
| Trauma | 0.00 | 0.03 | 0.18 | .859 |  | 0.00 | 0.03 | 0.18 | .859 |
| DV: Credulity |  |  |  |  |  |  |  |  |  |
| Trauma | 0.18 | 0.03 | 7.33 | < .001 |  | 0.18 | 0.03 | 7.33 | < .001 |
|  |  |  |  | |  |  |  |  | |
|  | β | *SE* | 95% CI | |  | β | *SE* | 95% CI | |
| Indirect effects |  |  |  | |  |  |  |  | |
| Total indirect effect | 0.03 | 0.01 | 0.02, 0.05 | |  | 0.05 | 0.01 | 0.03, 0.07 | |
| Trust | 0.00 | 0.00 | 0.00, 0.00 | |  | 0.00 | 0.00 | -0.01, 0.01 | |
| Mistrust | 0.00 | 0.00 | 0.00, 0.00 | |  | 0.00 | 0.00 | 0.00, 0.01 | |
| Credulity | 0.03 | 0.01 | 0.02, 0.05 | |  | 0.05 | 0.01 | 0.03, 0.07 | |

*Note.* All models were conducted with a subsample of *n* = 1 535 participants. Epistemic trust, mistrust, and credulity were assessed with the ETMCQ^11^. The ITQ^55^ was used to assess symptoms of PTSD and disturbances in self-organization (DSO). The models adjusted for age, gender, education, and income as covariates of non-interest. All coefficients are standardized. Indirect effects were computed with bootstrapping using 5 000 iterations.

**Supplementary Table 8**

*Moderated Mediation Results of Relational Trauma Through Epistemic Stance on PTSD and DSO by Age Group*

|  | PTSD | | | |  | DSO | | | |
| --- | --- | --- | --- | --- | --- | --- | --- | --- | --- |
|  | β | 95% CI | *t* | *p* |  | β | 95% CI | *t* | *p* |
| DV: Trust |  |  |  |  |  |  |  |  |  |
| Intercept | 1.14 | 0.35, 1.93 | 2.83 | .005 |  | 1.14 | 0.35, 1.93 | 2.84 | .005 |
| Relational Trauma | -1.20 | -2.18, -0.23 | -2.43 | .015 |  | -1.24 | -2.21, -0.27 | -2.51 | .012 |
| Age Group | -0.51 | -0.92, -0.10 | -2.46 | .014 |  | -0.52 | -0.93, -0.11 | -2.48 | .013 |
| Relational Trauma:Age Group | 0.60 | 0.09, 1.10 | 2.33 | .020 |  | 0.61 | 0.11, 1.11 | 2.40 | .017 |
| DV: Mistrust |  |  |  |  |  |  |  |  |  |
| Intercept | -0.91 | -1.76, -0.06 | -2.10 | .036 |  | -0.89 | -1.74, -0.05 | -2.07 | .039 |
| Relational Trauma | 1.07 | 0.02, 2.11 | 2.01 | .045 |  | 1.06 | 0.02, 2.09 | 2.01 | .045 |
| Age Group | 0.52 | 0.08, 0.96 | 2.31 | .021 |  | 0.50 | 0.06, 0.94 | 2.24 | .025 |
| Relational Trauma:Age Group | -0.60 | -1.14, -0.06 | -2.17 | .030 |  | -0.59 | -1.13, -0.05 | -2.16 | .031 |
| DV: Credulity |  |  |  |  |  |  |  |  |  |
| Intercept | -0.90 | -1.72, -0.07 | -2.13 | .033 |  | -0.88 | -1.70, -0.06 | -2.10 | .036 |
| Relational Trauma | 1.14 | 0.13, 2.15 | 2.21 | .028 |  | 1.11 | 0.11, 2.12 | 2.17 | .030 |
| Age Group | 0.53 | 0.11, 0.96 | 2.46 | .014 |  | 0.52 | 0.09, 0.94 | 2.39 | .017 |
| Relational Trauma:Age Group | -0.60 | -1.12, -0.07 | -2.23 | .026 |  | -0.57 | -1.09, -0.05 | -2.17 | .031 |
| DV: PTSD/ DSO |  |  |  |  |  |  |  |  |  |
| Intercept | 9.77 | 9.25, 10.28 | 37.14 | < .001 |  | 9.07 | 8.63, 9.51 | 40.56 | < .001 |
| Relational Trauma | 0.46 | -0.16, 1.09 | 1.45 | .148 |  | 0.26 | -0.27, 0.79 | 0.96 | .339 |
| Trust | -0.27 | -0.57, 0.03 | -1.75 | .080 |  | -0.51 | -0.76, -0.25 | -3.88 | < .001 |
| Mistrust | -0.19 | -0.54, 0.16 | -1.04 | .297 |  | 0.44 | 0.14, 0.73 | 2.86 | .004 |
| Credulity | 1.21 | 0.85, 1.57 | 6.67 | < .001 |  | 1.24 | 0.94, 1.55 | 8.04 | < .001 |

*Note.* Parameters are presented for each regression path of the two moderated mediation models predicting PTSD and DSO, respectively. The first three regressions reflect the a-paths, i.e., effects of relational trauma, age group, and their interaction on the three epistemic stance dimensions (epistemic trust, mistrust, and credulity), which serve as dependent variables (DV). The last regression includes the coefficients of the b- and c-paths, i.e., direct effects of relational trauma and the epistemic stance dimensions on PTSD or DSO. Epistemic trust, mistrust, and credulity were assessed with the ETMCQ^11^. The ITQ^55^ was used to assess symptoms of PTSD and disturbances in self-organization (DSO). All models were conducted with a subsample of *n* = 838 participants. Parameters were determined using Model 7 in PROCESS^65^.

**Supplementary Note 3**

***Exploratory Analysis: Sequential Path Model***

Based on theoretical considerations after preregistration, we tested a sequential model where trauma nature predicts DSO through epistemic stance (moderated by age at trauma) and DSO, in turn, predicts the severity of PTSD symptoms.

The direct effect of relational trauma on PTSD was not significant (β = 0.39, 95% CI [-0.16, 0.94], *t*(832) = 1.40, *p* = .163), neither was the indirect effect of relational trauma on PTSD through DSO (β = 0.15, 95% CI [-0.16, 0.44]).

When looking at conditional effects, the indirect effect of relational trauma on PTSD through Trust and DSO was significant if trauma occurred in childhood (β = 0.17, 95% CI [0.02, 0.36]), but not significant if trauma occurred during adulthood (β = 0.00, 95% CI [-0.04, 0.05]). The corresponding moderated mediation effect was also significant (IMM = -0.16, 95% CI [-0.37, -0.02]).

Similarly, the indirect effect of relational trauma on PTSD through Credulity and DSO was significant if trauma occurred in childhood (β = 0.39, 95% CI [0.02, 0.79]), but not significant if trauma occurred during adulthood (β = -0.02, 95% CI [-0.14, 0.09]). The corresponding moderated mediation effect was also significant (IMM = -0.42, 95% CI [-0.83, -0.04]).

However, the indirect effect of relational trauma on PTSD through Mistrust and DSO was not significant irrespective of whether trauma occurred during childhood (β = 0.12, 95% CI [-0.03, 0.34]) or adulthood (β = -0.03, 95% CI [-0.09, 0.01]).

**Supplementary Figure 1**

*Sequential Moderated Mediation of Relational Trauma on PTSD Through Epistemic Stance by Age Group and DSO*

*Note.* The model was conducted with a subsample of *n* = 835 participants. Epistemic trust, mistrust, and credulity were assessed with the ETMCQ^11^. The ITQ^55^ was used to assess symptoms of PTSD and disturbances in self-organization (DSO). Parameters were determined using a custom model in PROCESS^65^. Coefficients are standardized. * *p* < .05; ** *p* < .01; *** *p* < .001.
